# Salvianolic acid B promotes angiogenesis and inhibits cardiomyocyte apoptosis by regulating autophagy in myocardial ischemia

**DOI:** 10.1186/s13020-023-00859-w

**Published:** 2023-11-28

**Authors:** Qi Chen, QingYang Xu, Huilin Zhu, Junyi Wang, Ning Sun, Huimin Bian, Yu Li, Chao Lin

**Affiliations:** 1 Department of Physiology and Pathophysiology, State Key Laboratory of Medical Neurobiology, School of Basic Medical Sciences, Fudan University, 138 Yi Xue Yuan Road, Shanghai, 200032 People’s Republic of China; 2 Wuxi School of Medicine, Jiangnan University, Wuxi, 214013 China; 3 School of Medicine and Holistic Integrative Medicine, Nanjing University of Chinese Medicine, Nanjing, 210023 China; 4 School of Pharmacy, Nanjing University of Chinese Medicine, Nanjing, 210023 China; 5 Jiangsu Key Laboratory for Pharmacology and Safety Evaluation of Chinese Materia Medica, Nanjing University of Chinese Medicine, Nanjing, Xianlin Avenue, Qixia District, 210023 China

**Keywords:** Myocardial ischemia, Angiogenesis, Apoptosis, Salvianolic acid B, Autophagy

## Abstract

**Background:**

Myocardial ischemia (MI) can cause angina, myocardial infarction, and even death. Angiogenesis is beneficial for ensuring oxygen and blood supply to ischemic tissue, promoting tissue repair, and reducing cell damage. In this study, we evaluated the effects of Salvianolic acid B (Sal B) against myocardial ischemia and explored its underlying mechanism on autophagy.

**Methods:**

The anti-apoptosis effect of Sal B was conducted by staining Annexin V-FITC/PI and Hoechst as well as evaluating apoptosis bio-markers at protein level in H9c2 cells at glucose deprivation condition. HUVECs were co-cultured with H9c2, and the tube formation assay was used to monitor Sal B’s impact on angiogenesis. The MI model of mice was induced by intraperitoneal injection of isoproterenol (ISO). The effect of Sal B on MI mice was evaluated by HE, Masson, immunohistochemistry, WB and kits. In addition, Atg5 siRNA was applied to verify whether the protective effect of Sal B was regulated to autophagy.

**Results:**

In H9c2, Sal B reduced the levels of lactate dehydrogenase (LDH), malondialdehyde (MDA) and reactive oxygen species (ROS), improved the levels of superoxide dismutase (SOD) and mitochondrial membrane potential, downregulated the expressions of Bax and cleaved-Caspase3, upregulated the expression of Bcl-2. Therefore, Sal B could significantly inhibit the damage of H9c2 caused by glucose deprivation. In the co-culture system of H9c2 and HUVECs, vascular endothelial growth factor (VEGF) level in the supernatant was dramatically raised by Sal B. Sal B upregulated the expressions of VEGF, platelet derived growth factor (PDGF) and endothelial marker CD31. It implied that Sal B exerted a significant pro-angiogenic effect. Moreover, Sal B increased the expression of LC3, Atg5, and Beclin1, while reducing the level of P62. When the expression of Atg5 was inhibited, the protective effects of Sal B on apoptosis and angiogenesis was reversed.

**Conclusions:**

Sal B inhibited cardiomyocyte apoptosis and promoted angiogenesis by regulating autophagy, thereby improving MI.

## Background

Myocardial ischemia (MI) is a disease that seriously threatens human health, and its incidence has been on the rise in recent years [1]. Persistent ischemia and hypoxia lead to myocardial infarction, accompanied by ventricular remodeling, cardiac insufficiency and heart failure [2]. MI results from inadequate blood flow and nutrient deprivation brought on by coronary artery stenosis or spasm. In acute and chronic MI, the establishment of angiogenesis or collateral circulation helps to ensure the oxygen and blood supply of the ischemic tissue, promotes the repair of damaged tissues, and maintains the heart function [3]. Therefore, promoting myocardial angiogenesis is important for the prevention and treatment of MI.

Cardiomyocyte apoptosis is a programmed cell death that occurs actively under pathological factors, and it is an important mechanism for MI [4]. Cardiomyocytes are terminally differentiated cells and lack the ability to regenerate, so it is significant to inhibit myocardial cell apoptosis. Cardiomyocyte function and structure are maintained in large part by autophagy [5]. Cardiomyocytes can produce adenosine triphosphate (ATP) through autophagy to greatly alleviate the energy crisis caused by ischemia. In addition, activating autophagy promotes angiogenesis [6].

**Fig. 1:**
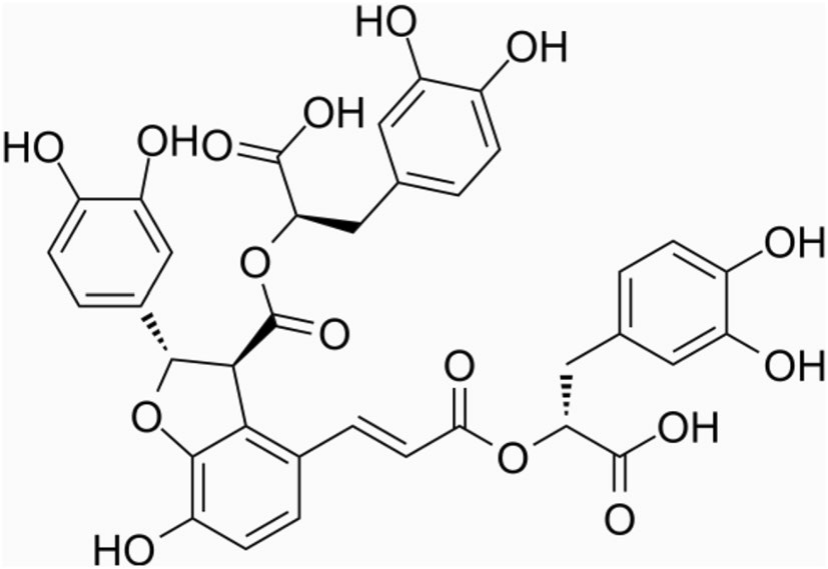
The chemical structure of Sal B

In recent years, Chinese medicines have shown great effects on treating MI [7–9]. Chinese herbal medicine’s saponins work as a preventative measure against cardiac ischemia-reperfusion injury [10]. Numerous Chinese herbal medications, such as Berberine, Schisandrin B, Resveratrol, et al., can block the unfolded protein response-related signaling pathways to avoid oxidative stress damage and apoptosis via the induction of ER stress in myocardial I/R [11]. The Traditional Chinese Medicine *Salvia miltiorrhiza* has been proved to dilate coronary arteries, improve microcirculation and reduce myocardial oxygen consumption [12]. Salvianolic acid B (Sal B, Fig. 1) is the main active ingredient in *Salvia Miltiorrhiza* that exerts various pharmacological effects [13, 14]. Sal B reduced MI injury by inhibiting the SIRT1-AMPK-PGC-1 signaling pathway’s activation of the NLRP3 inflammasome [15]. Sal B mitigated MI impairment through stimulating mitophagy and suppressing NLRP3 inflammasome activation [16]. During the treatment of cardiovascular diseases, it is found that Sal B has a promoting effect on angiogenesis in the ischemic area. Sal B could not only reduce the area of MI, but also promote angiogenesis in the ischemic myocardium of rats [17]. However, no research has yet examined how Sal B affects angiogenesis and cell death simultaneously in MI. In this research, we intended to observe the effects of Sal B on angiogenesis and cardiomyocyte apoptosis, and explored whether the mechanism was related to autophagy.

## Methods

### Reagents

Sal B (HPLC ≥ 98%) was obtained from Chengdu PureChem Standard Co., Ltd. (Chengdu, China). Isoproterenol was purchased from Shanghai Sine Pharmaceutical Co., Ltd. (Shanghai, China). Elisa kits (VEGF and PDGF) were purchased from Multi-Sciences (Lianke) Biotech Co., Ltd. (Hangzhou, China), and other kits (LDH, SOD, MDA, AST, CK, Ca^2+^, NO, eNOS and NOS) were purchased from Jiancheng Biotech Co., Ltd. (Nanjing, China). Each antibody was bought from Abcam (Cambridge, UK).

### Administration of drugs with the myocardial ischemia model

Twenty male ICR mice, weighing 20–25 g each, were acquired from Nanjing University’s Nanjing Biomedical Research Institute. All mice were kept in an environment that was consistent with a humidity level of 40 ± 5%, a 12 h light cycle, and an average temperature of 23 ± 1 °C. Mice were given free access to the normal diet and water. The study received formal endorsement from the Institutional Ethics Committee for Animal Care (A171002) and adhered to the prescribed Guidelines and Policies for Animal Surgical Procedures promulgated by Nanjing University of Chinese Medicine during the execution of the animal studies. Mice were divided into four groups: Control + Con siRNA, ISO + Con siRNA, ISO + Sal B + Con siRNA, ISO + Sal B + Atg5 siRNA. The mice were given Atg5 siRNA by the tail vein injection once a week for two weeks. One week later, the administration group mice were injected intraperitoneally with Sal B (10 mg/kg) [18, 19] for 7 days. On the 5th day of administration, the myocardial ischemia model was constructed by intraperitoneal injection of ISO (3 mg/kg), once a day for 3 days [20]. The electrocardiogram changes of mice were recorded through the Power lab data collection and analysis system.

### Histological analysis

The hearts were weighed before being fixed in 10% buffered formalin. Slices of the ventricle that were 2 mm thick and subsequently sliced into 4 μm pieces were fixed in paraffin. Hematoxylin and eosin (HE) and Masson were employed to stain the sections, and five randomly chosen fields on each section were picked to be examined.

### Examinations for biology

Blood was drawn and left at room temperature for two hours. Centrifugation at 3000 rpm for 10 min was used to produce serum, which was then chilled to − 80 °C. Elisa kits were utilized to measure serum levels of PDGF and VEGF. Chemichromatometry was employed to quantify the levels of LDH, SOD, MDA, AST, CK, NO, NOS, and eNOS, as well as Ca^2+^, in accordance with kit instructions.

### Immunohistochemistry

Atg5 polyclonal antibody (1:100) was initially applied to the aorta slices and incubated for 2 h at 37 °C. After washing the slices, the samples were incubated with the secondary antibody (1:100) for 30 min at room temperature, followed by 5 min with 0.5 g/L diaminobenzidine.

### Cell culture and grouping

HUVECs and H9c2 cell lines were obtained from the Chinese Academy of Science (Shanghai, China). The Dulbecco’s modified Eagle’s medium (DMEM) with 10% fetal bovine serum (FBS) and 1% benzylpenicillin/streptomycin was used to cultivate cells in the sixth and twelfth passages. The high sugar medium was replaced with sugar free medium to replicate the glucose deprivation model. At the same time, Sal B was given, and relevant indicators were tested 24 h later.

### Cell viability testing

Serum-starved for 16 h after reaching 60% confluence, H9c2 cells (1 × 10^4^/well) were sown in a 96-well microplate. Following the treatment, 20 µL of 2,5-diphenyl-2 H-tetrazolium bromide (MTT) solution was added to each well. The plate was then left to incubate for 4 h. Ultimately, the absorbance was detected at 570 nm.

### Hoechst staining

H9c2 were pre-treated for 24 h before being planted in 24-well plates at a density of 5 × 10^4^ cells per well. Following the procedure, the adhered cells were washed three times in PBS after removing the culture medium. After that, cells were stained for 5 min at 37 °C with Hoechst 33,258. The apoptotic alterations of cells were seen and captured on camera using a fluorescent microscope following three PBS washes.

### Flow cytometry

Following the manufacturer’s instructions, a double fluorescence staining methodology was implemented to recognize early apoptosis and necrosis via staining Annexin V-FITC/PI. A flow cytometer (FACS Calibur, BD Biosciences, San Jose, CA) was utilized to measure the fluorescence for each individual.

### Mitochondrial membrane potential (MMP)

H9c2 were pretreated for 24 h prior being seeded in 24-well plates at a density of 5 × 10^4^ cells per well. Afterwards treatment, cells were stained with JC-1 (Beyotime, Shanghai, China) following compliance with the manufacturer’s instructions for the MMP test kit.

### Wound healing assay

The cells were seeded at a density of 2 × 10^5^ cells per well. When the cells grew to 80% of the well, the supernatant was removed, and a 1 mL sterile pipette tip was used to make a scratch along the drawn line. After scratching, PBS was used three times to wash the plate. The cells were administered for 24 h. After the drug intervention, the supernatant was removed, and a standard optical microscope was used to take pictures.

### Tube formation assay

After added VEGF-A and Sal B to HUVECs (3 × 10^3^ cells/well), the cells were seeded into 96-well culture plates precoated with Matrigel™ Matrix Growth Factor Reduced (BD Biosciences, NJ, USA) at 37 °C for 6 h [21]. The tube-like structures were observed and photographed under a phase-contrast inverted microscope (Leica Microsystems GmbH) at a 4× magnification. Five randomly selected fields per culture plate well were applied to compute the tube length.

### Immunofluorescence

After permeabilization with 0.5% Triton X-100 and fixing with 4% paraformaldehyde, H9c2 cells were utilized and blocked with 1% BSA. Then, the cells were incubated with primary antibodies at 4 °C overnight and secondary antibody at room temperature for 1 h. Lastly, ZEN 2011 imaging software was employed to take images of the fluorescence expression via a Zeiss inverted microscope.

### Western blot analysis

The BCA assay kit (Beyotime, Shanghai, China) was used to determine the protein concentration in accordance with the guidelines. Subsequent to the 10% SDS-PAGE separation, 30 µg of proteins were transferred to PVDF membranes, and the primary antibodies were subjected to an overnight incubation at 4 °C following blocking. After that, membranes were incubated with the secondary antibody at room temperature for 90 min. The identification of the target proteins was ultimately accomplished utilizing an ECL system from Millipore (Millipore, MA, USA), and the results were visualized through employment of a ChemiDoc XRS system from Bio-Rad (Bio-Rad, CA, USA).

#### Statistical analysis

One-way ANOVA and Tukey’s multiple comparison tests were employed to evaluate the data via Prism (Version 6.0; GraphPad Software Inc.). Data were displayed as the mean ± SEM. P values less than 0.05 were recognized as statistically significant.

## Results

### Sal B promoted the angiogenesis after glucose deprivation

**Fig. 2:**
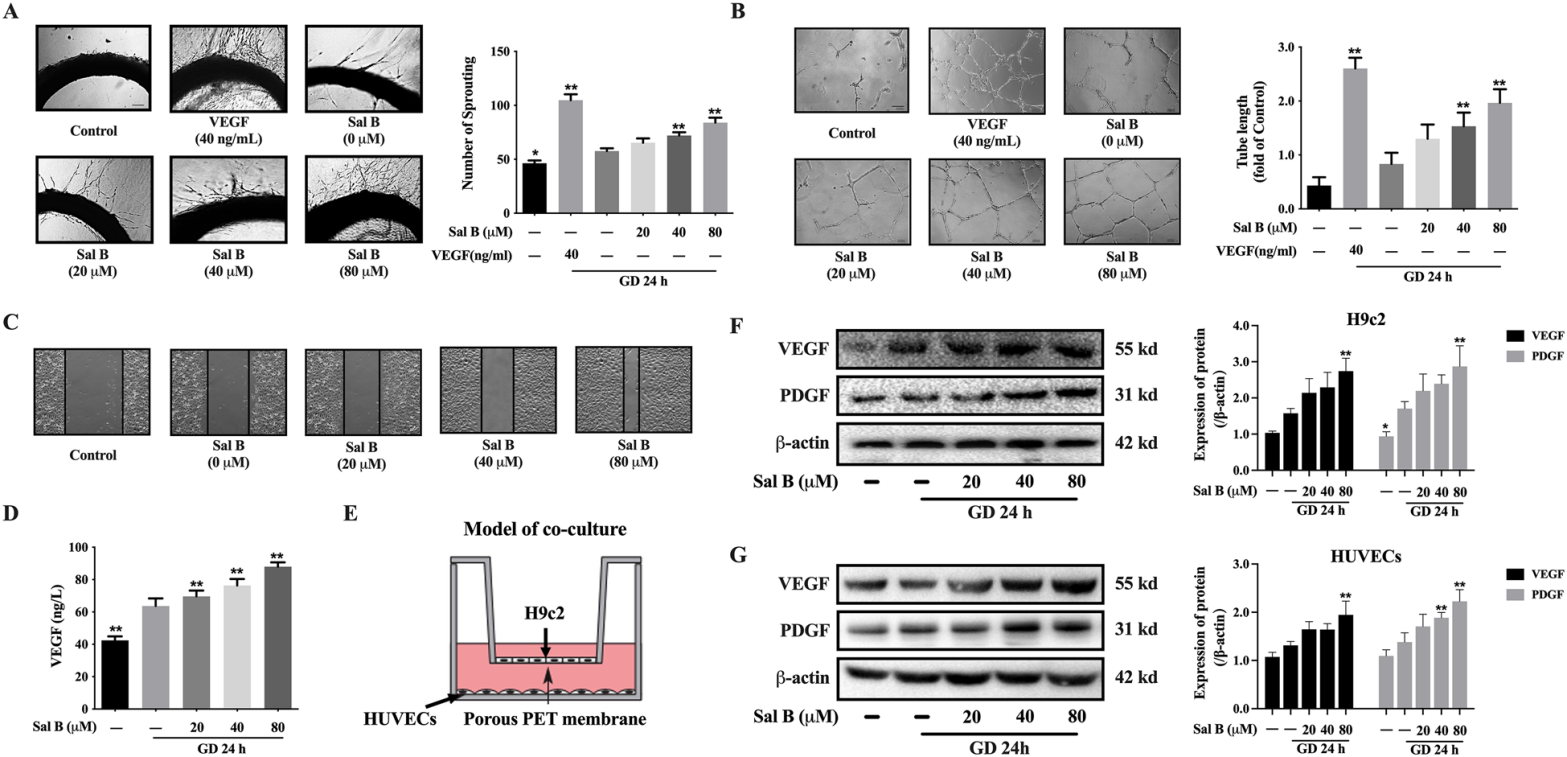
Sal B promoted the angiogenesis after glucose deprivation. **A** Sal B increased the angiogenesis in rats (4×, n = 4); **B** Sal B promoted the tubule formation in HUVECs (4×, n = 5); **C** Sal B facilitated the migration of HUVECs (4×, n = 5); **D** Sal B boosted the secretion of VEGF (n = 5); **E** The model of co-culture; **F** Sal B increased the expressions of VEGF and PDGF in H9c2 (n = 3); **G** Sal B increased the expressions of VEGF and PDGF in HUVECs (n = 3); Data are shown as the mean ± SD. *P < 0.05, **P < 0.01, compared to GD 24 h without Sal B group

In a glucose free environment, the cultured rat arterial rings exhibited significant angiogenesis. As the concentrations of Sal B increased, the number of sprouting gradually increased. While, VEGF exhibits the strongest angiogenic effect (Fig. 2A). Likewise, Sal B promoted the tube-formation in HUVECs in a manner dependent on dose (Fig. 2B). Wound healing assay showed the deprivation of glucose accelerated the migration of HUVECs, and the treatment of Sal B facilitated the migration of HUVECs further (Fig. 2C). Then, we treated H9c2 with Sal B and detected the secretion of VEGF in supernatant. The content of VEGF significantly increased in the glucose free environment, and Sal B stimulated the secretion of VEGF in a dose-dependent manner. HUVECs were co-cultured with H9c2, the structure diagram was shown in Fig. 2E. The expressions of VEGF and PDGF were elevated by 80 µM Sal B both in H9c2 (Fig. 2F) and HUVECs (Fig. 2G).

### The deprivation of glucose aggravated the apoptosis of H9c2

**Fig. 3:**
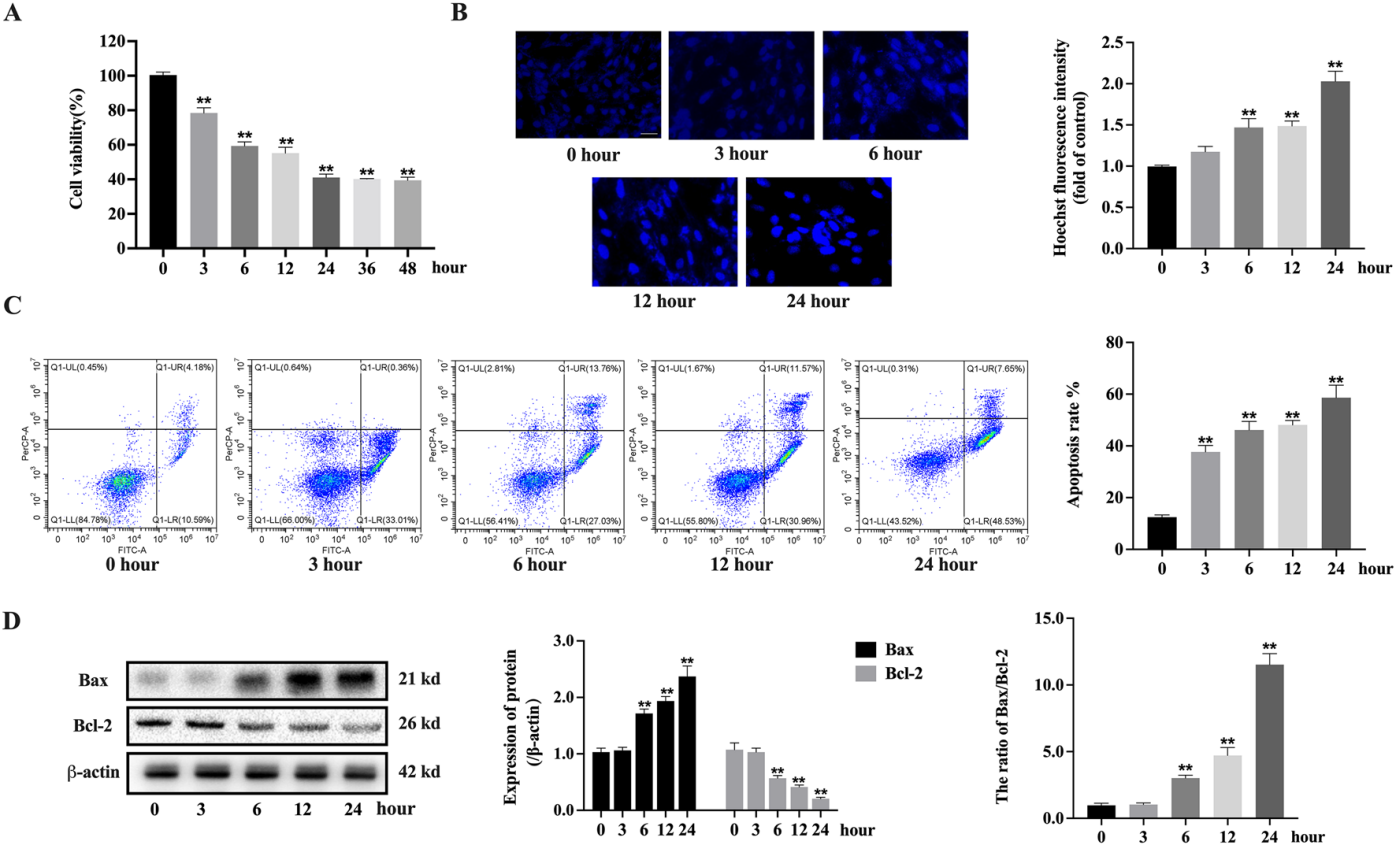
The deprivation of glucose aggravated the apoptosis of H9c2. **A** Effects of glucose deprivation at different time points on cell viability of H9c2 (n = 6); **B** Hoechst staining showed the apoptosis of H9c2 (20×, n = 3); **C** Flow cytometry revealed apoptosis of H9c2 (n = 3); **D** WB assayed the expressions of Bax and Bcl-2 (n = 3); Data are shown as the mean ± SD. *P < 0.05, **P < 0.01, compared to GD 0 h group

Glucose deprivation significantly reduced the cell viability of H9c2, and the cell viability decreased to a minimum of 40% at 24 h (Fig. 3A). Hoechst staining showed that after glucose deprivation for more than 6 h, the number of apoptotic nuclei increased dramatically (Fig. 3B). After 3 h of glucose deprivation, a number of cells were dramatically increased at early apoptosis condition. When glucose deprivation reached 6 and 12 h, the early apoptosis of cells was significantly reduced, while the late apoptosis was significantly increased. When the glucose deprivation time reached 24 h, the late apoptosis of cells further increased (Fig. 3C). The result illustrated the expressions of Bax and Bcl-2 did not alter after glucose deprivation for 3 h. After 6 h of glucose deprivation, the pro-apoptotic protein Bax gradually increased with time, while the anti-apoptotic protein Bcl-2 shortened with time. The ratio of Bax to Bcl-2, an indicator of cell apoptosis, significantly increased when glucose deprivation for 24 h (Fig. 3D).

### Sal B alleviated H9c2’s apoptosis brought on by glucose restriction

**Fig. 4:**
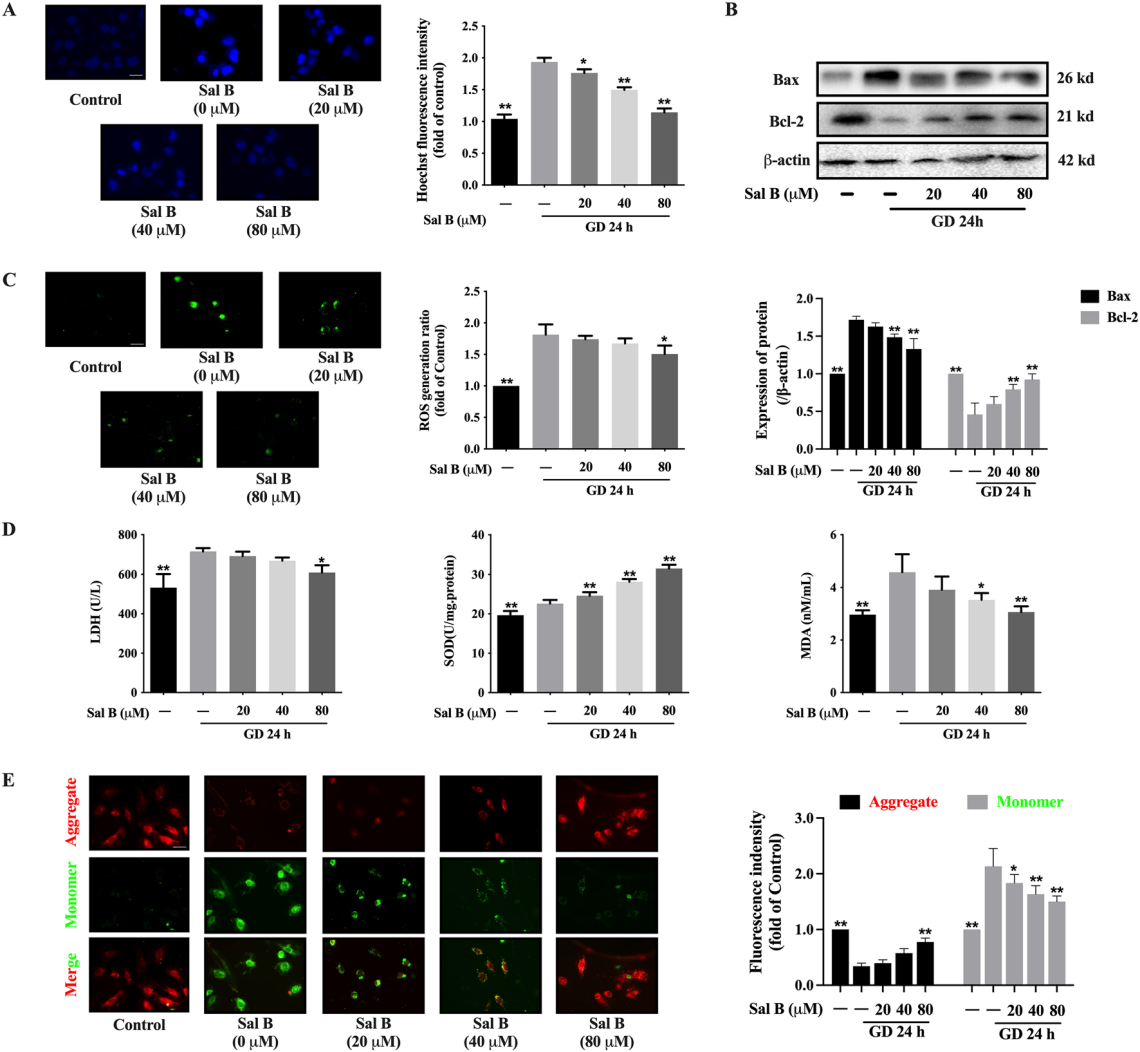
Sal B alleviated the apoptosis of H9c2 induced by glucose deprivation. **A** Hoechst staining showed Sal B reduced the apoptosis of H9c2 (20×, n = 3); **B** WB showed Sal B regulated the expressions of Bax and Bcl-2 (n = 3); **C** Immunofluorescence staining showed Sal B reduced the level of ROS (20×, n = 3); **D** Sal B reduced the content of LDH, SOD and MDA (n = 6); **E** JC-1 staining showed Sal B elevated the mitochondrial membrane potential (20×, n = 3); Data are shown as the mean ± SD. *P < 0.05, **P < 0.01, compared to GD 24 h without Sal B group

Sal B was administered in various amounts to treat H9c2 apoptosis that was brought on by glucose deprivation for 24 h. Sal B reduced the quantity of apoptotic cells in a dose-dependent manner, according to Hoechst staining (Fig. 4A). 40 and 80 µM Sal B dramatically raised the expression of Bcl-2 in response to glucose restriction while significantly decreasing the expression of Bax (Fig. 4B). The generation of oxygen free radicals is one of the primary underlying mechanisms during myocardial injury. The treatment with 80 µM Sal B could reduce the content of ROS in H9c2 induced by glucose deprivation (Fig. 4C). Correspondingly, 80 µM Sal B significantly reduced the content of LDH, and downregulated the levels of SOD and MDA in a dose-dependent manner (Fig. 4D). The decrease of mitochondrial membrane potential (MMP) is the earliest change of cell apoptosis. JC-1 staining showed the MMP of H9c2 was notably lowered after glucose deprivation for 24 h. While, Sal B elevated the MMP in a way that depends on dose (Fig. 4E).

### Sal B promoted the autophagy of H9c2 induced by glucose deprivation

**Fig. 5:**
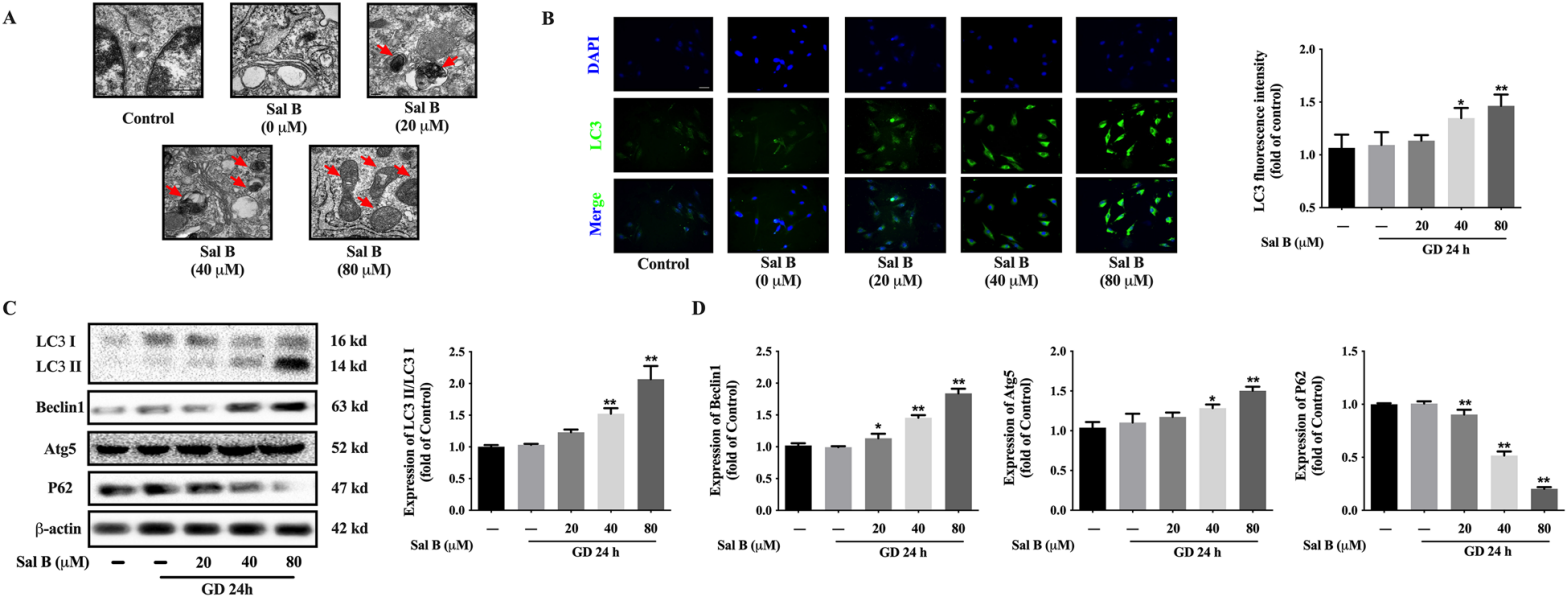
Sal B promoted the autophagy of H9c2 induced by glucose deprivation. **A** Electron microscopy showed Sal B promoted the formation of autophagosome (Scale bar = 0.2 μm, n = 3); **B** Immunofluorescence staining showed Sal B increased the expression of LC3 (20×, n = 3); **C**, **D** WB showed Sal B regulated the expressions of LC3, Beclin1, Atg5 and P62 (n = 3); Data are shown as the mean ± SD. *P < 0.05, **P < 0.01, compared to GD 24 h without Sal B group

The electron microscopy results showed that obvious autophagosomes appeared after administration of Sal B, and damaged mitochondria could also be seen in typical autophagosomes. Moreover, the number of autophagosomes ascended with the increase of Sal B dose (Fig. 5A). Immunofluorescence assay indicated that Sal B at 40 and 80 µM significantly raised the fluorescence intensity of LC3 (Fig. 5B). Similarly, 40 and 80 µM Sal B significantly improved the expression of LC3 II/LC3 I. Sal B also exerted a sharp upregulation effect on the expressions of autophagy specific proteins Beclin1 and Atg5. Sal B, however, dose-dependently reduced the expression of P62 (Fig. 5C).

### Sal B regulated the angiogenesis and apoptosis through Atg5

**Fig. 6:**
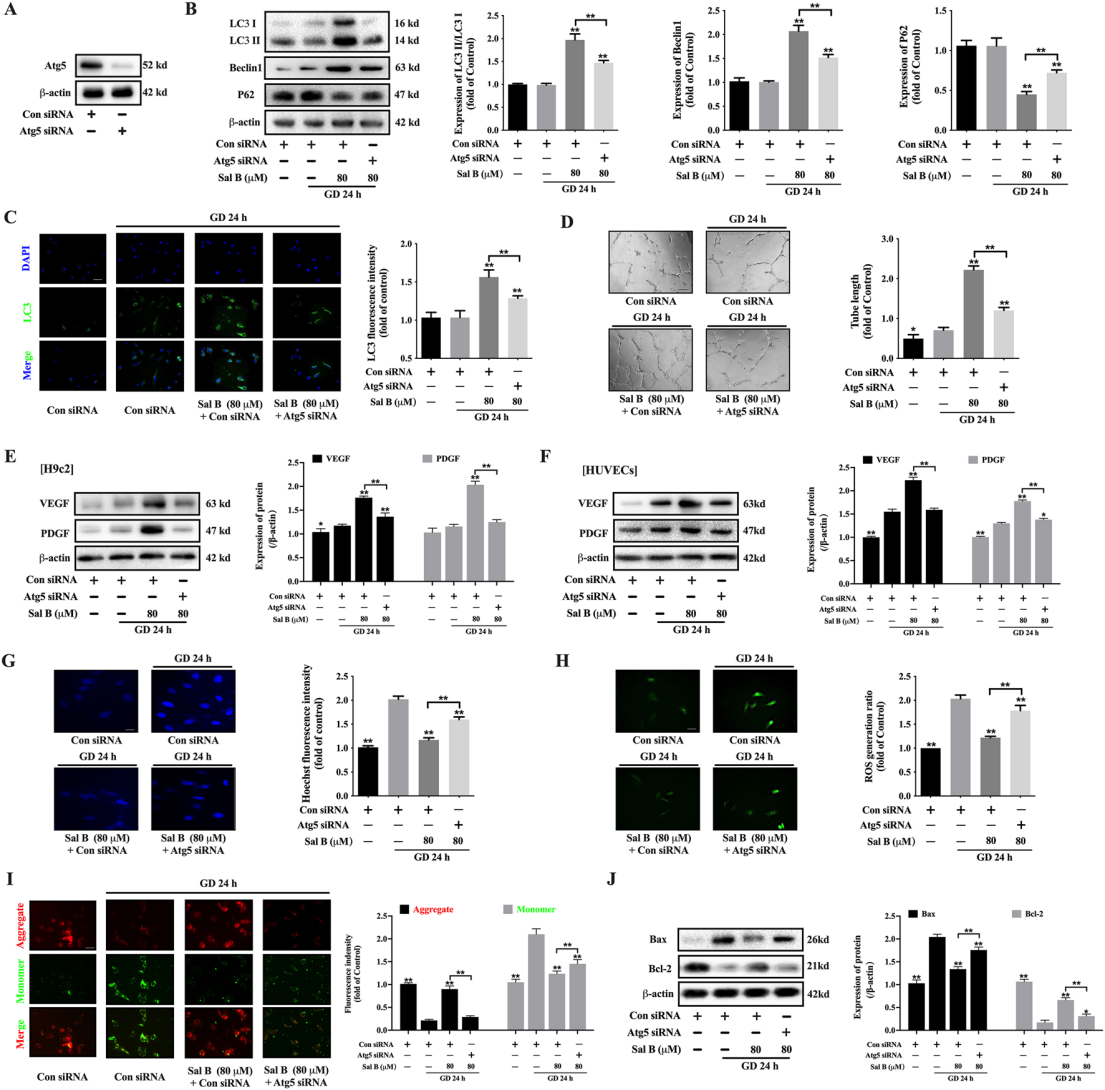
Sal B regulated the angiogenesis and apoptosis through Atg5. **A** The expression of Atg5 was inhibited by Atg5 siRNA (n = 3); **B** With the transfection of Atg5 siRNA, WB showed the effects of Sal B on regulating the expressions of LC3, Beclin1, Atg5 and P62 (n = 3); **C** Immunofluorescence staining showed the effect of Sal B on increasing the expression of LC3 was reduced by Atg5 siRNA (20×, n = 3); **D** The effect of Sal B on promoting the tubule formation in HUVECs was alleviated by Atg5 siRNA (4×, n = 5); **E** The effect of Sal B on increasing the expressions of VEGF and PDGF in H9c2 was alleviated by Atg5 siRNA (n = 3); **F** The effect of Sal B on increasing the expressions of VEGF and PDGF in HUVECs was alleviated by Atg5 siRNA (n = 3); **G** Hoechst staining showed the effect of Sal B on reducing the apoptosis of H9c2 was alleviated by Atg5 siRNA (20×, n = 3); **H** Immunofluorescence staining showed the effect of Sal B on reducing the level of ROS was alleviated by Atg5 siRNA (20×, n = 3); **I** JC-1 staining showed the effect of Sal B on elevating the mitochondrial membrane potential was reduced by Atg5 siRNA (20×, n = 3); **J** With the transfection of Atg5 siRNA, WB showed the effects of Sal B on regulating the expressions of Bax and Bcl-2 (n = 3); Data are shown as the mean ± SD. *P < 0.05, **P < 0.01, compared to GD 24 h without Sal B group

WB results confirmed that the expression of Atg5 in H9c2 was successfully inhibited by Atg5 siRNA (Fig. 6A). The effect of Sal B on increasing the expression of LC3 II/LC3 I and Beclin was significantly weakened by Atg5 siRNA (Fig. 6B, C). On the contrary, compared to the Sal B group transfected with control siRNA, Atg5 siRNA obviously increased the expression of P62 (Fig. 6B). HUVECs was also transfected with Atg5 siRNA, and Sal B’s ability to encourage angiogenesis was severely decreased (Fig. 6D). Both in H9c2 and HUVECs, Atg5 siRNA impaired the ability of Sal B to increase the expressions of VEGF and PDGF (Fig. 6E, F). In H9c2, Sal B significantly decreased the amount of apoptotic cells in H9c2, but was reversed by Atg5 siRNA (Fig. 6G). Likewise, Atg5 siRNA upregulated the content of ROS reduced by Sal B (Fig. 6H). JC-1 staining showed Atg5 siRNA downregulated MMP of H9c2 improved by Sal B (Fig. 6I). Compared to the Sal B group transfected with control siRNA, Bax expression was considerably upregulated and Bcl-2 expression was significantly downregulated by Atg5 siRNA. (Fig. 6J). It implied that Sal B regulated the angiogenesis and apoptosis of H9c2 through Atg5.

### Sal B improved isoproterenol (ISO) induced myocardial ischemia in mice through Atg5

**Fig. 7:**
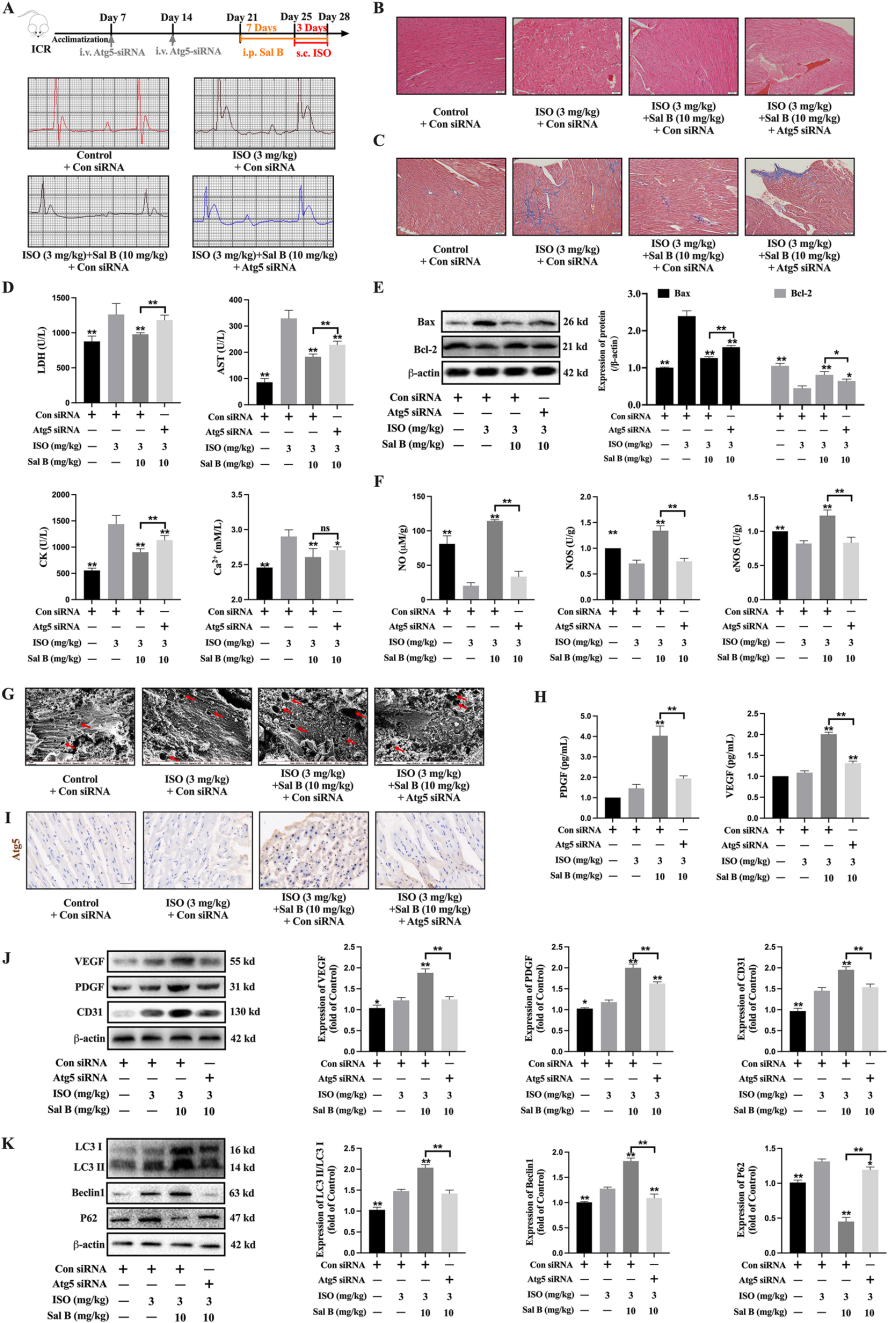
Sal B improved ISO induced myocardial ischemia in mice through Atg5. **A** Overall flow chart of animal experiment and changes in mice electrocardiogram. **B** Analysis of myocardial injury by HE staining (Scale bar = 50 μm, n = 5); **C** Analysis of myocardial fibrosis by Masson staining (Scale bar = 50 μm, n = 5); **D** The levels of LDH, AST, CK and Ca^2+^ were discovered using ELISA kits (n = 5); **E** The expressions of Bax and Bcl-2 in mice myocardial tissue were assayed by WB (n = 3); **F** The levels of NO, NOS, and eNOS were detected by ELISA kits (n = 5); **G** The angiogenesis pores on the surface of myocardial tissue with the use of scanning electron microscopy (Scale bar = 2 μm, n = 3); **H** ELISA kits were used to measure the levels of PDGF and VEGF in the serum (n = 5); **I** The expression of Atg5 in mice myocardial tissue was detected by immunohistochemistry (n = 3); **J** The expressions of VEGF, PDGF, and CD31 in mice myocardial tissue were detected by WB (n = 3); **K** The expressions of LC3 I, LC3 II, Beclin1 and P62 in mice myocardial tissue were detected by WB (n = 3); Data are shown as the mean ± SD. *P < 0.05, **P < 0.01, compared to ISO with control siRNA group

The myocardial ischemia in mice was induced by intraperitoneal injection of ISO, the flow chart was shown in Fig. 7A. The result illustrated that the electrocardiogram (ECG) of mice changed significantly after ISO injection, which confirmed the successful establishment of MI model. Sal B clearly suppressed the infiltration of inflammatory cells and decreased the fibrosis of cardiac tissue, as seen by the HE and Masson stains. However, the improvement effect of Sal B was weakened after Atg5 siRNA transfection (Fig. 7B, C). Sal B significantly downregulated the levels of LDH, AST, CK and Ca^2+^ in serum of MI mice. Mice treated with Sal B and Atg5 siRNA exerted higher levels of LDH, AST, and CK than mice treated with Sal B and control siRNA. (Fig. 7D). Sal B reduced the expression of Bax and increased the expression of Bcl-2 in MI mice, while the anti-apoptosis effect of Sal B was blocked by Atg5 siRNA (Fig. 7E). It implied that Sal B improved MI mice induced by ISO through Atg5.

### Sal B promoted angiogenesis in myocardial ischemia mice through Atg5

Sal B also significantly upregulated the content of vascular endothelial markers NO, NOS, and eNOS in MI mice, but the effect was reversed when mice were co-treated with Atg5 siRNA (Fig. 7F). Scanning electron microscopy analysis revealed that myocardial tissue surface angiogenic holes were dramatically enhanced by Sal B. When Atg5 was inhibited, the number of angiogenesis pores decreased significantly (Fig. 7G). The results of immunohistochemistry confirmed that Atg5 siRNA the expression of Atg5 in myocardial tissue (Fig. 7I). Both in serum and myocardial tissue, Sal B promoted the content and expressions of PDGF, VEGF and CD31. However, the increase caused by Sal B were reversed by Atg5 siRNA (Fig. 7H-J). In addition, the increase of LC3 II/I and Beclin 1 and the decreased of P62 caused by Sal B were reversed by Atg5 siRNA (Fig. 7K). It suggested that Sal B promoted angiogenesis in MI mice through Atg5-mediated autophagy.

## Discussion

MI is a common type of cardiovascular disease and has become one of the largest hidden killers of cardiovascular disease [22]. Therefore, the prevention and treatment of this disease is particularly important. In this study, the myocardial ischemia model was constructed by intraperitoneal injection of ISO. ISO can excite β1 receptor, which increases myocardial excitability, contraction, heart rate, metabolism, and promotes myocardial oxygen consumption, resulting in cardiac overload. At the same time, it can also excite β2 receptors, causing peripheral vascular dilation, reduce cardiac blood flow, lower arterial pressure, and decrease coronary flow. Compared to surgical ligation, the advantage of ISO is that it is easy to operate and does not require special equipment. The disadvantage is that it is unable to accurately locate the infarcted area [23].

Salvia miltiorrhiza, a traditional Chinese medicine, possesses the capacity to ameliorate pain, disperse blood stasis, and facilitate the stimulation of meridians and blood circulation [24]. Cardiovascular illnesses have benefited greatly from its use. It has been reported that *Salvia miltiorrhiza* possesses salvianolic acid as its most abundant water-soluble ingredient, while Sal B is the most prevalent and biologically active component of salvianolic acid [25]. Salvianolic acid not only provides protection against myocardial ischemia-reperfusion injury [26], but also improves MI and myocardial cell hypertrophy. In this study, we confirmed that Sal B inhibited myocardial cell apoptosis and promoted angiogenesis by upregulating autophagy activity.

Angiogenesis is essentially defined as the proliferation or migration of vascular endothelial cells based on preexisting capillaries and/or microvessels, culminating in the production of new capillaries in the form of sprouting or non-sprouting from blood vessels [27]. Vascular regeneration in ischemic myocardium requires endothelial cells to continuously divide, migrate, and undergo a series of processes such as adhesion and reconnection, ultimately forming new capillary lumens [28]. VEGF is one of the potent and highly specific proangiogenic factors. VEGF targets endothelial cells, which can increase endothelial cell mitosis, promote vascular endothelial cell proliferation, differentiation, and angiogenesis [29]. The expression of VEGF is relatively low in normal hearts, while it is significantly increased in ischemic myocardial cells. However, the endogenous increase in VEGF is relatively brief and insufficient to establish sufficient collateral circulation, which cannot meet the blood supply of ischemic myocardium [30]. The administration of exogenous VEGF in the ischemic myocardial infarction area can promote angiogenesis, thereby improving collateral circulation [31]. In addition, as an important mitogenic factor, PDGF has the ability to stimulate cell division and proliferation [32]. It was found that Sal B could not only reduce the area of MI, but also promote the vascular density of ischemic and infarcted areas. Our results found that Sal B upregulated the expressions of VEGF and PDGF in MI, promoted the migration of HUVECs and luminal formation, and increased the sprouting of aortic rings. It indicated that Sal B could improve blood supply by promoting angiogenesis.

Myocardial cell apoptosis is an active and orderly programmed death that occurs under pathological factors, and is an important mechanism for the occurrence of MI. Myocardial cells are terminal cell lines and do not have the ability to divide and regenerate [33]. Therefore, reversing myocardial cell damage and avoiding apoptosis is particularly significant. Ischemia and hypoxia are the main factors that stimulate myocardial cell apoptosis [34]. The characteristic structural changes of cell apoptosis are mainly concentrated in the nucleus, manifested as nuclear shrinkage. Bcl-2 is a major regulatory gene for cell apoptosis [35]. When apoptosis signals stimulate cells, the pro-apoptotic protein Bax shifts from the cytoplasm to the outer membrane of mitochondria. Bax then goes through polymerization, creating membrane holes that let Cytochrome C out of the mitochondria and into the cytoplasm. As Caspase-3 is activated in the cytoplasm, Cytochrome C can cause cell death [36]. Our results indicated that the initial phase of apoptosis occured in H9c2 after 3 h of glucose deprivation, reaching a peak at 6 h. Cells in the early stage of apoptosis gradually decreased at 24 h, but the total number of apoptotic cells has increased significantly. Treatment with Sal B reversed the nuclear shrinkage induced by glucose deprivation, enhanced the expression of Bcl-2, and downregulated the expressions of Bax and Cleaved-Caspase3, thereby inhibiting myocardial cell apoptosis. Consistent with reported literature, Sal B diminished the cardiomyocytes loss in acute myocardial infarction [37].

Enhanced generation of oxygen radicals and lipid peroxidation can exacerbate myocardial damage [38]. SOD can inhibit the damage caused by oxygen radicals, eliminate toxic superoxide, and protect cells from radical damage. The amount of SOD activity is a proxy for the organism’s capacity to neutralize oxygen radicals. MDA is usually used to demonstrate the level of lipid peroxidation, indicating the degree of damage to cells by oxygen radicals [39]. During MI, mass oxygen radicals are produced in the myocardial tissue, and the ability to clear free radicals is also reduced. The disruption of the balance between the generation and clearance of ROS can result in a large accumulation of oxygen radicals, leading to serious damage to myocardial tissue [40]. The degree of damage caused by oxygen radicals can be indirectly evaluated by changes in MDA content and the activity of SOD. The results suggested that ischemic injury reduced SOD activity and raised MDA levels in myocardial tissue, indicating an enhancement in radical production and a decrease in clearance ability during ischemia. Sal B intervention alleviated the above changes, and thus alleviated the damage to ischemic myocardium. Oxidative stress and autophagy are closely related. By removing molecules and organelles harmed by oxidative stress, autophagy can play a role in the control of redox metabolism. Nevertheless, antioxidant cell defense pathways can be used to modulate autophagy [41]. But more investigation is required to discover whether Sal B can control oxidative stress via autophagy.

Autophagy, as the “gatekeeper” of intracellular environmental homeostasis, plays a significant role in clearing damaged mitochondria and alleviating cell damage [42]. Most studies have shown that many stress responses after MI can activate autophagy. Autophagy can provide energy for cells by degrading necrotic or senescent organelle, thus maintaining intracellular homeostasis [43]. The complex formed by Beclin-1 and PI3KC3 is involved in the formation and expansion of phagocytic vesicles, which is an important indicator of autophagy initiation [44]. At the same time, two ubiquitination protein systems are also activated: Atg12-Atg5-Atg16 system and Atg8/LC3 system [45]. The complex of Atg12-Atg5-Atg16 adheres to phagocytic vesicles in the form of tetramers and promotes the expansion of phagocytic vesicles [46]. Upon the synthesis of the LC3 precursor, LC3 I is generated under the catalysis of Atg4, a cytoplasmic constituent. Subsequently, Atg7 initiates the activation of LC3 I, facilitating its conjugation with phosphatidylethanolamine alcohol to give rise to LC3 II. Following the fusion of autophagosomes and lysosomes, LC3 II is subject to degradation by hydrolytic enzymes. It is noteworthy that the quantity of LC3 II exhibits a direct correlation with the number of autophagosomes, rendering it a pivotal indicator for the assessment of autophagic activity. [47]. The level of P62 is related to the damage of ubiquitinated protein degradation. The excessive accumulation of p62 reflects the obstacle of protein clearance and the obstruction of autophagy [48]. The study of autophagy tide can reflect the role of autophagy in ischemic injury.

Autophagy and apoptosis are both independent and interrelated: on the one hand, autophagy can occur before apoptosis and transform into apoptosis; as opposed to that, autophagy can inhibit apoptosis and maintain cell survival [49]. The conclusions on the relationship between autophagy and apoptosis are not unified. Some researchers believe that drugs inhibit apoptosis by inhibiting autophagy, while others think that drugs exert anti-apoptotic effects by promoting autophagy [50]. The results of our study showed that after administration of Sal B, autophagy lysosomes increased. Under the electron microscope, autophagy lysosomes and degraded organelle fragments in lysosomes could be clearly observed. Meanwhile, WB results indicated that Sal B upregulated the expressions of LC3II/I, Beclin1, and Atg5, while inhibiting the expression of P62. Therefore, Sal B not only upregulated autophagy activity, but also maintained the integrity of autophagy tide.

Can autophagy regulate angiogenesis under stress conditions such as myocardial ischemia and hypoxia? It has been reported that autophagy promoted the formation of tubular structures in bovine aortic endothelial cells (BAECs) [51]. Hypoxia reduced the nuclear localization of high mobility group protein B1 (HMGB1) in skeletal muscle cells, promoted the release of HMGB1 and autophagy, thereby promoting angiogenesis [52]. However, other viewpoints suggested that autophagy could inhibit angiogenesis. It was reported that human plasmin K5 reduced the ability of endothelial cells to form tubes by inducing the expression of Beclin1 [53]. Although the above research conclusions were different and did not confirm the specific role of moderate autophagy in angiogenesis, it can be confirmed that autophagy is an important regulator of angiogenesis. Our study found that Sal B promoted angiogenesis by upregulating autophagy activity, indicating that autophagy played a significant role in Sal B mediated angiogenesis. Sal B’s precise target was not determined by us, and subsequent studies will examine the interaction between H9c2 and HUVECs.

## Conclusion

We found that Sal B could prevent cell death and promote angiogenesis by regulating autophagy, therefore improving MI. Studies have confirmed that various cardiovascular disorders are protected against by Sal B, such as MI and atherosclerosis. The mechanism of Sal B in regulating cardiovascular disease has been revealed, which offers the theoretical underpinning for the promotion of the use of Sal B in healthcare.

## Data Availability

The datasets used and/or analyzed during the current study are available from the corresponding author on reasonable request.
